# Correlations between the expression of molecules in the TGF-β signaling pathway and clinical factors in adamantinomatous craniopharyngiomas

**DOI:** 10.3389/fendo.2023.1167776

**Published:** 2023-10-03

**Authors:** Lu Jin, Kefan Cai, WenTao Wu, Youchao Xiao, Ning Qiao, Fangzheng Liu, Siming Ru, Lei Cao, Haibo Zhu, Jiwei Bai, Chunhui Liu, Chuzhong Li, Peng Zhao, Yazhuo Zhang, Songbai Gui

**Affiliations:** ^1^ Department of Neurosurgery, Beijing Tiantan Hospital, Capital Medical University, Beijing, China; ^2^ Beijing Neurosurgical Institute, Capital Medical University, Beijing, China

**Keywords:** adamantinomatous craniopharyngiomas, hypothalamus invasion, TGF-β signaling pathway, β-catenin, endocrinopathy, prognosis

## Abstract

**Objective:**

To investigate the clinical and pathological factors associated with preoperative hypothalamus invasion and postoperative outcomes of adamantinomatous craniopharyngiomas (ACPs) after the expanded endonasal approach (EEA) resection.

**Methods:**

Ninety-three specimens of ACPs, consisting of 71 primary and 22 recurrent tumors, were investigated for the expression of TGF-β1, SMAD2, SMAD3, and β-catenin by immunohistochemistry staining. The clinical information of relevant patients, including the extent of resection, hypothalamus invasion, endocrinopathy, complications, and prognosis, was reviewed. The relationships between the expression of these immunopathological markers and clinical factors were analyzed.

**Results:**

Endocrinological dysfunctions were more common in recurrent patients and primary patients with hypothalamus invasion in the comparisons. For recurrent patients, the rate of gross total resection (GTR) was significantly lower than for primary patients (63.6% vs. 90.1%, *P* = 0.007). According to radiological and intraoperative findings, invasive ACPs (IACPs) included 48 (67.6%) cases in primary tumors. The expression of TGF-β1 and β-catenin was significantly higher in recurrent tumors (*P* = 0.021 and *P* = 0.018, respectively) and IACPs (*P* = 0.008 and *P* = 0.004, respectively). The expression level of TGF-β1 was associated with hypothalamus involvement (Puget grade, *P* = 0.05; Vile grade, *P* = 0.002), postoperative endocrinopathy (*P* = 0.01), and pituitary stalk preservation (*P* = 0.008) in primary patients. In addition, the extent of resection, treatment history, hypothalamic invasion, and level of TGF-β1 expression had significant influences on tumor recurrence/progression after surgery separately.

**Conclusion:**

Our study demonstrated the potential role of TGF-β1 in the regulation of hypothalamus invasion in ACPs and the prediction of prognosis after EEA surgery. The TGF-β signaling pathway may represent a crucial mechanism in the aggressive behavior and progression of ACPs.

## Introduction

Craniopharyngiomas (CPs) are benign epithelial tumors of the sellar region, accounting for 1%–15% of all primary intracranial neoplasms (1). They originate from Rathke’s pouch epithelium and can be histologically distinguished into two variants: adamantinomatous CP (ACP) and papillary CP (PCP) (2). Although they are regarded as histologically benign (WHO grade I) neoplasms, CPs, especially ACPs, are often biologically invasive and tend to infiltrate the parasellar neurovascular structures (e.g., the pituitary stalk, optic chiasma, and hypothalamus), which makes surgery for CPs extremely challenging and risky accompanied by confusing recurrence even after gross total resection (GTR) (3–5).

The clinical symptoms of CPs include visual acuity or field deficit, headache, growth arrest, signs of panhypopituitarism, hydrocephalus, and neuropsychological disturbances, depending on the location and expansion toward adjacent structures (6). The treatment of CPs includes surgery and radiotherapy (7). For years, neurosurgeons proposed a variety of classifications to describe their location, fitting the needs to imply corresponding transcranial surgical approaches. Recently, the expanded endonasal approach (EEA) has been introduced as an ideal alternative to craniotomy via the corridor between the optic chiasm and pituitary gland, with a higher rate of resection and decreased postoperative morbidity (8, 9). In order to define the relationship between CPs and the pituitary–hypothalamus in the context of EEA, Pascual and colleagues proposed schemes defining the integrity and distortion of the third ventricle floor (TVF) and hypothalamus by estimating the mammillary body angle ranges (10). Moreover, Puget et al. also developed a feasible preoperative classification system to assess hypothalamic involvement caused by primary CPs, which classified the morphological distortions of the hypothalamus into three grades (11).

Pathological studies of ACPs have consistently demonstrated that their aggressive behavior is characterized by the presence of finger-like epithelial cells arranged in lobules protruding into adjacent tissues, surrounded by a florid glial and inflammatory reactive tissue (12). Other hallmarks indicating regressive alterations include calcification, cholesterol clefts, and anuclear ghost cells (also known as “wet keratin”) as well as cell clusters with nuclear β-catenin accumulation (12, 13). These cluster cells are often arranged in whirl-like structures and represent an alternatively differentiated population with WNT pathway activation, evidenced by the expression of target genes (e.g., *LEF1* and *AXIN2*) (14, 15). Insights into the functional significance of the β-catenin accumulating cells have suggested that these clusters act as signal “hubs,” secreting a plethora of growth factors and cytokines (e.g., SSH, FGFs, BMPs, TGF-β1, IL1, IL6) (16–18), which activate specific pathways in nearby non-accumulating tumor cells, driving tumor infiltration into surrounding tissues in an autocrine/paracrine manner (19, 20).

The transforming growth factor-β (TGF-β) signaling pathway is activated in many tumors and involved in numerous cellular processes including proliferation, differentiation, migration, invasion, and epithelial–mesenchymal transition (21, 22). Recently, pieces of evidence have demonstrated that the TGF-β signaling pathway can also interact with the Wnt/β-catenin signaling pathway, which contributes to tumor progression and invasion by sequentially activating the downstream effectors of TCF/LEF transcription factors (21, 23, 24). Up to now, few studies have investigated the expression levels of the TGF-β signaling pathway in ACPs, and the molecular mechanisms mediating invasion and recurrence also remain undefined. We assumed that hypothalamus invasion of ACPs may be correlated with the TGF-β signaling pathway. Then, we used the immunohistochemistry technology to examine the expression levels of key molecules in the TGF-β signaling pathway (TGF-β1, SMAD2, and SMAD3). We attempted to analyze the relationships between the expression of molecules in the TGF-β pathway and β-catenin in ACPs and examine its associations with clinical factors and treatment outcomes.

## Methods

### Patient selection

A total of 93 ACPs (22 recurrent ACPs and 71 primary ACPs) formaldehyde-fixed specimens collected between April 2017 and June 2022 were available for this study. The corresponding clinical files of these patients were searched in the Electronic Medical Record of the Department of Neurosurgery, Beijing Tiantan Hospital, Capital Medical University. The inclusion criteria were as follows: 1) ACP confirmed by pathological report, 2) without treatment history of radiotherapy, 3) positive and clear immunohistochemical staining, 4) complete medical records, and 5) resection via EEA. This study was approved by the Ethics Committee of Beijing Tiantan Hospital (approval number KY 2021-041-02), and written informed consent was obtained from all patients.

### Neuroimaging and classification

**Figure 1:**
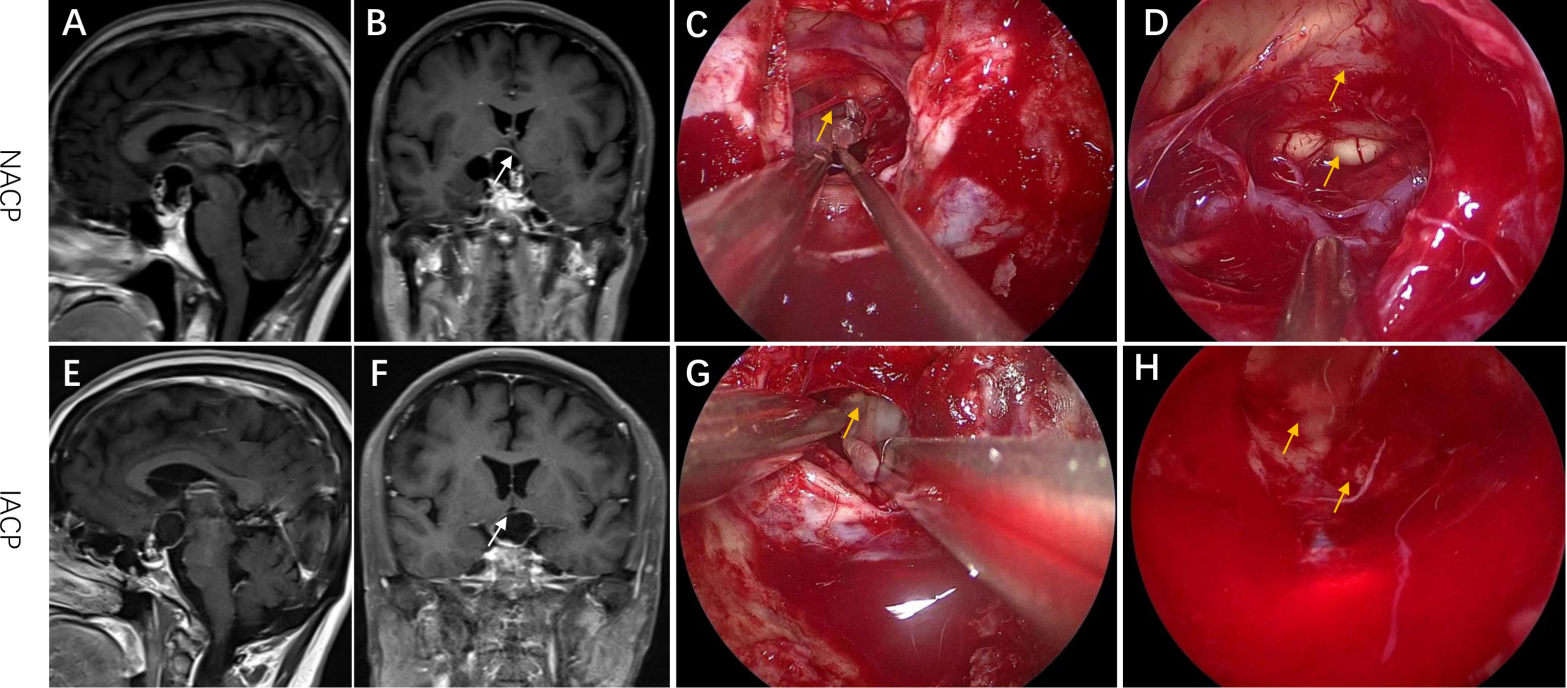
Representative radiological and intraoperative images of IACP and NACP. **(A, B)** Sagittal and coronal MRI images of NACP. The arrow pointed to the site of TVF. **(C, D)** The intraoperative images of EEA surgery for NACP. **(C)** The arrow pointed to the interface between the hypothalamus and the tumor capsule. **(D)** The arrow indicated the TVF and mammillary bodies. **(E, F)** Sagittal and coronal MRI images of IACP. The arrow pointed to the site of TVF. **(G, H)** The intraoperative images of EEA surgery for IACP. **(G)** The arrow pointed to the connected plane between the hypothalamus and the tumor capsule. **(H)** The arrow indicated the TVF. EEA, expanded endonasal approach; TVF, third ventricle floor; NACP, non-invasive adamantinomatous craniopharyngioma; IACP, invasive adamantinomatous craniopharyngioma.

**Figure 2:**
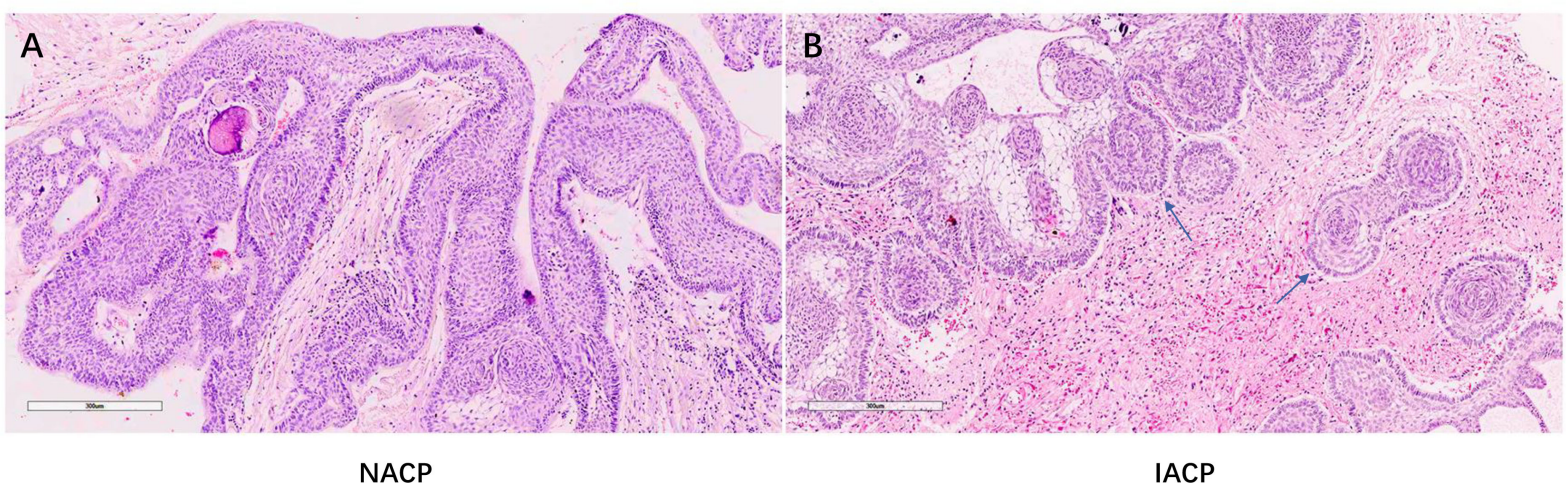
**(A, B)** Representative HE staining pathological images of NACP and IACP (×200). **(B)** The arrow indicated the finger-like epithelial protrusion into adjacent brain tissues. NACP, non-invasive adamantinomatous craniopharyngioma; IACP, invasive adamantinomatous craniopharyngioma.

We divided the primary ACPs into two types. Type I referred to non-invasive ACPs (NACPs), which were restricted to the suprasellar compartment with compression of the hypothalamus or not on sagittal and coronal MRI (**Figures 1A, B**) but without invasion into the TVF confirmed by intraoperative videos (**Figures 1C, D**) and hematoxylin and eosin (HE) staining of tumor specimens (**Figure 2A**). Type II was invasive ACPs (IACPs). The tumor–hypothalamus cleavage plane is not identifiable on sagittal and coronal MRI (**Figures 1E, F**), and splitting between the interface cannot be achieved by sharp or blunt dissection (**Figure 1G**). HE staining indicated hypothalamus invasion (**Figure 2B**), with TVF open at different degrees after the operation (**Figure 1H**).

Radiographic features were obtained by MR and CT scans. The tumor volume (TV) was calculated by a standardized A*B*C/2 method in which variables were measured by the longest diameter in each plane. Postoperative images were compared with preoperative ones to determine the extent of tumor resection, which can be classified into three categories: GTR (no residual tumor on postoperative images), subtotal resection (STR, >95% resection), and partial resection (PR, <95%). In the statistical analysis, the latter two were combined into one group of non-gross total resection (NTR) in this study. Follow-up MR imaging was scheduled at 3 months after surgery and then at regular intervals of 6–12 months. Recurrence/progression referred to the new neoplasms after GTR or enlarged residuals after NTR.

### Immunohistochemical staining and analysis

Formalin-fixed, paraffin-embedded ACP specimens were used to perform immunohistochemical staining (IHC) with the two-step plus poly-horseradish peroxidase (HRP) method. Briefly, 4-μm-thick sections were cut from each tissue block using a rotary microtome (RM2135, Leica, California, USA) and placed on poly(L-lysine)-coated slides. The slides were deparaffinized, rehydrated, immersed in 10 mm of Tris/EDTA buffer (pH 9.0), pretreated in a microwave oven for 20 min, and then rinsed for 15 min with phosphate-buffered saline (PBS). After blocking with 3% hydrogen peroxide for 10 min at room temperature, the slides were incubated at 4°C overnight with primary antibodies: anti-TGF-β1 (dilution 1:500, rabbit monoclonal antibody, ab215715, Abcam, Cambridge, UK), anti-SMAD3 (dilution 1:1,000, rabbit monoclonal antibody, ab40854, Abcam), anti-SMAD2 (dilution 1:100, rabbit monoclonal antibody, ab40855, Abcam), and anti-β-catenin (dilution 1:500, rabbit monoclonal antibody, ab32572, Abcam). The slides were then stained with the two-step plus Poly-HRP Anti-Rabbit IgG Detection System (PV-9001; ZSGB-Bio, Beijing, CN). After visualization of the reaction with mixed DAB (ZLI-9017; ZSGB-Bio) for 4 min, the slides were counterstained with hematoxylin for 3 min and covered with a glycerin gel. For a negative control, the primary antibody was replaced by phosphate-buffered saline.

### Aperio Digital IHC analysis and quantification

Immunostained slides were scanned by a Leica Aperio AT2 scanner (at ×400 magnification) and analyzed using a Leica Aperio ImageScope v12.3.0.5056. Before running the macros procedure, a classifier for identifying the areas of interest (tumor regions) was created by the algorithm of Genie to improve the accuracy of the results by excluding the stromal elements. The Cytoplasmic v2 algorithm was chosen for the automatic scoring of each antibody, which estimates the stains as negative (0), weak (1+), moderate (2+), and strong (3+) according to the scoring criteria threshold. SMAD2, SMAD3, and β-catenin, due to the biologically activated forms, are translocated into the nuclei, and we include the estimated nuclear results for the subsequent analyses. For TGF-β1, the region of interest was the cytoplasm. Finally, the H-score of each slide was calculated by the formula: H-score = 1 × (percentage of weak staining) + 2 × (percentage of moderate staining) + 3 × (percentage of strong staining) (25–27). Based on the mean value of each biomarker’s H-score, patients were divided into two groups: 1) high expression, H-score ≥ mean value, and 2) low expression, H-score < mean value, separately.

### Endocrinological evaluation

Pre- and postoperative endocrinological status was evaluated by checking the hormone levels of prolactin, GH axis, thyroidal axis, adrenal axis, and gonadal axis. Endocrinological dysfunctions were categorized according to specified pituitary hormonal axes, including hyperprolactinemia, hypothyroidism, hypoadrenalism, hypogonadism, or GH deficiency. Meanwhile, diagnosis for diabetes insipidus (DI) was based on polydipsia and polyuria, urine-specific gravity, and urine osmolarity.

### Surgical procedure and follow-up

All patients underwent an expanded EEA via infrachiasmatic corridor with the tuberculum sellae removed. After opening the arachnoid membrane and releasing the cerebrospinal fluid (CSF), a wide exposure of the sellar and suprasellar area was achieved. Intracapsular debulking was carried out with the combination of sharp and blunt dissection, carefully protecting the pituitary stalk and hypothalamus as much as possible. The cleavage plane of the CP–hypothalamus interface was dissected along the gliosis tissue for invasive ones. After removing the tumor content and capsule, reconstruction of the skull base was performed in a standard fashion, with a tailored biological membrane (Beijing Tianxinfu Medical Appliance Corporation, Beijing, CN) and a prepared vascularized nasoseptal flap placed onto the skull base to cover the dura defect tightly.

### Statistical analysis

Parametric comparison analyses were used for continuous variables, and a chi-square (*χ* ^2^) test was used to compare categorical variables. Analyses were conducted by SPSS (IBM, CA, USA), with the significant difference alpha level set at *P <*0.05 for all tests. The Kaplan–Meier method was used for progression-free survival (PFS) analysis. GraphPad Prism version 8 (GraphPad Software, La Jolla, CA, USA) was used to plot the graphs and curves.

## Results

### Patient data

The characteristics of 93 patients with ACPs who underwent EEA surgery are summarized in **Table 1**. There were 48 female and 45 male patients with a mean age of 38.1 ± 16.3 years. Twenty-two patients had a history of surgery before this admission. The common complaints included visual impairment (76.3%), headache (36.6%), dizziness (5.4%), amenorrhea (14.0%), polyuria/polydipsia (23.7%), vomiting (10.8%), somnolence (11.8%), memory disturbance (30.1%), and behavioral instability (8.6%). Among these clinical manifestations, only memory disturbance showed a significant difference between the primary and recurrent patients (*P* = 0.001). The mean duration of the symptoms was 7.8 ± 7.4 months.

**Table 1:** Clinical characteristics of patients with primary/recurrent ACPs.

Characteristic	Total (93)	Primary (71)	Recurrent (22)	*P*-value
Gender (female)	48 (51.6%)	39 (54.9%)	9 (40.9%)	0.330
Age (mean ± SD, years)	38.1 ± 16.3	39.9 ± 15.2	31.9 ± 18.5	**0.044**
Presentation				
Visual impairment	71 (76.3%)	53 (74.7%)	18 (81.8%)	0.577
Headache	34 (36.6%)	27 (38.0%)	7 (31.8%)	0.801
Dizziness	5 (5.4%)	3 (4.2%)	2 (9.1%)	0.589
Amenorrhea	13 (14.0%)	12 (16.9%)	1 (4.6%)	0.288
Polyuria/polydipsia	22 (23.7%)	16 (22.5%)	6 (27.3%)	0.775
Vomiting	10 (10.8%)	7 (9.9%)	3 (13.6%)	0.696
Somnolence	11 (11.8%)	8 (11.3%)	3 (13.6%)	0.718
Memory disturbance	28 (30.1%)	15 (21.1%)	13 (59.1%)	**0.001**
Behavioral instability	8 (8.6%)	7 (9.9%)	1 (4.6%)	0.675
Duration (mean ± SD, months)	7.8 ± 7.4	8.3 ± 7.7	6.2 ± 6.2	0.335
Radiography				
Content (solid/cystic/mixed)	22/36/35	19/27/25	3/9/10	0.422
Calcification	62 (66.7%)	45 (63.4%)	17 (77.3%)	0.304
Hydrocephalus	10 (10.8%)	5 (7.0%)	5 (22.7%)	0.053
Tumor volume (mean ± SD, cm^3^)	14.3 ± 15.6	13.8 ± 14.8	15.5 ± 18.5	0.677
Preoperative endocrinopathy				
Hyperprolactinemia	45 (48.4%)	37 (52.1%)	8 (36.4%)	0.229
Hypothyroidism	32 (34.4%)	19 (26.8%)	13 (59.1%)	**0.009**
GH deficiency	16 (17.3%)	8 (11.3%)	8 (36.4%)	**0.019**
Hypoadrenalism	16 (17.2%)	4 (5.6%)	12 (54.6%)	**<0.001**
Hypogonadism	21 (22.6%)	11 (15.5%)	10 (45.5%)	**0.007**
Diabetes insipidus	22 (23.7%)	16 (22.5%)	6 (27.3%)	0.775
Preoperative BMI (kg/m^2^)	26.5 ± 5.2	24.9 ± 4.0	31.2 ± 5.6	**<0.001**
Extent of resection (GTR/NTR)	78/15	64/7	14/8	**0.007**
Pituitary preservation	24 (25.8%)	20 (28.2%)	4 (18.2%)	0.416
Recurrence/progression	17 (18.3%)	8 (11.3%)	9 (40.9%)	**0.035**
Postop persistent DI	26 (27.9%)	16 (22.5%)	10 (45.5%)	0.056

Bold values indicate significant differences of p < 0.05.

According to radiological and intraoperative findings, the tumor contents consisted of three morphological types: pure solid mass (22 cases), pure cystic mass (36 cases), and solid cystic mass (35 cases). In the primary tumors, type I (NACPs) included 23 (32.4%) cases, and type II (IACPs) included 48 (67.6%) cases. Focal calcification was found in 66.7% of all patients on CT scans, and 10 patients presented with hydrocephalus. The median volume of the tumor was 14.3 ± 15.6 cm^3^. There were no significant differences between these radiological features between the primary and recurrent groups of patients. However, compared with patients with primary tumors, the recurrent patients had higher levels of BMI (*P* < 0.001) and higher percentages of hypopituitarism. Specially, the overall percentage of preoperative hypopituitarism was 34.4% of hypothyroidism (26.8% vs. 59.1%, *P* = 0.009), 48.4% of hyperprolactinemia (52.1% vs. 36.4%, *P* = 0.229), 17.3% of growth hormone (GH) deficiency (11.3% vs. 36.4%, *P* = 0.019), 17.2% of hypoadrenalism (5.6% vs. 54.6%, *P* < 0.001), 22.6% of hypogonadism (15.5% vs. 45.5%, *P* = 0.007), and 23.7% of diabetes insipidus (DI) (22.5% vs. 27.3%, *P* = 0.775) (**Table 1**).

### Surgical outcomes and complications

GTR, STR, and PR were achieved in 78 (83.9%), 13 (14.0%), and 2 (2.1%) patients, respectively. For recurrent patients, GTR was achieved in 14 cases, which was significantly lower than the percentage of primary patients (63.6% vs. 90.1%, *P* = 0.007). The pituitary stalk was partly or completely preserved in 24 (25.8%) patients which showed no significant difference between primary and recurrent patients (28.2% vs. 18.2%, *P* = 0.416). Two patients experienced intracranial infection, and one had postoperative CSF leakage, which was repaired by autogenous broad fascia transplantation. Patients were followed up for 6–60 months with a median time of 23 months, and tumor recurrence or progression was found in 17 (18.3%) cases. During the follow-up, persistent DI was sustained in 26 (27.9%) patients who needed Minirin to control their urinary output. None of the patients died during the follow-up period (**Table 1**).

### Expression of TGF-β1, SMAD3, SMAD2, and β-catenin

The average H-scores of TGF-β1, SMAD3, SMAD2, and β-catenin were 41.8 (range: 4.4 to 159.5), 63.1 (range: 13.0 to 100.0), 77.8 (range: 29.4 to 107.7), and 84.1 (range: 22.2 to 202.1), respectively, and there was significantly greater TGF-β1 and β-catenin expression in the specimens from patients with a recurrent tumor (*P* = 0.021 and *P* = 0.018, respectively) (**Table 2**). Pearson correlation showed a significant correlation between the expression of β-catenin and TGF-β1 (*R* = 0.265, *P* = 0.01), as well as SMAD3 (*R* = 0.308, *P* = 0.003) (**Table 3**). For the primary cases, TGF-β1 and β-catenin expression levels were significantly greater in the group of patients with hypothalamic invasion (*P* = 0.008 and *P* = 0.004, respectively) (**Table 4**).

**Table 2:** Comparison of immunohistochemical characteristics between patients with primary and recurrent ACPs.

Biomarker	Total	Primary	Recurrent	*P*-value
TGF-β1	41.75 ± 22.77	38.33 ± 20.02	52.79 ± 37.51	**0.021**
SMAD3	63.11 ± 23.20	62.51 ± 22.98	65.04 ± 24.34	0.657
SMAD2	77.81 ± 15.72	77.98 ± 15.98	77.27 ± 15.20	0.855
β-catenin	84.10 ± 36.31	79.17 ± 32.50	99.99 ± 43.69	**0.018**

Bold values indicate significant differences of p < 0.05.

**Table 3:** Correlations among H-scores for the key biomarkers assessed by immunohistochemical staining.

Biomarkers	Value	TGF-β1	SMAD3	SMAD2	β-Catenin
TGF-β1	*R*		0.163	0.027	0.265^*^
	*P*		0.118	0.798	**0.010**
SMAD3	*R*	0.163		0.089	0.308^*^
	*P*	0.118		0.394	**0.003**
SMAD2	*R*	0.027	0.089		0.015
	*P*	0.798	0.394		0.887
β-Catenin	*R*	0.265^*^	0.308^*^	0.015	
	*P*	**0.010**	**0.003**	0.887	

R, Pearson’s correlation coefficient; P, significance level.

Bold values indicate significant differences of p < 0.05. *, with significant correlation by p < 0.05.

**Table 4:** Comparison of immunohistochemical characteristics between patients with/without hypothalamus invasion in primary ACPs.

Biomarker	NACPs	IACPs	*P*-value
TGF-β1	29.31 ± 15.76	42.64 ± 20.53	**0.008**
SMAD3	59.54 ± 23.54	63.93 ± 22.83	0.456
SMAD2	79.35 ± 15.43	75.10 ± 17.06	0.298
β-Catenin	63.37 ± 22.04	86.75 ± 34.14	**0.004**

Bold values indicate significant differences of p < 0.05.

### Factors related to pre- and postoperative endocrinopathy

**Figure 3:**
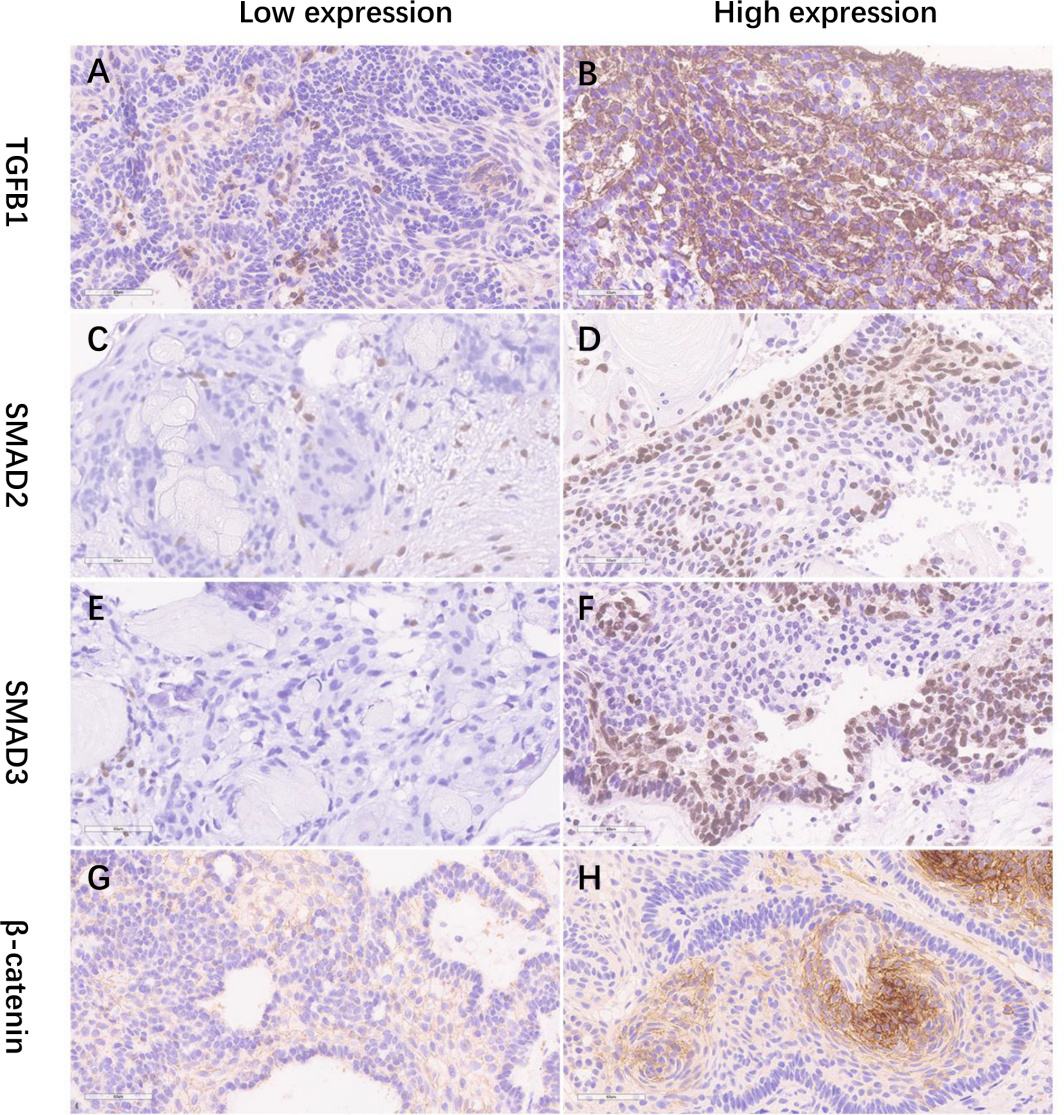
**(A–H)** The IHC staining illustrations of the low and high expression of TGF-b1, SMAD2, SMAD3, and b-catenin (×400). IHC, immunohistochemical staining.

**Figure 4:**
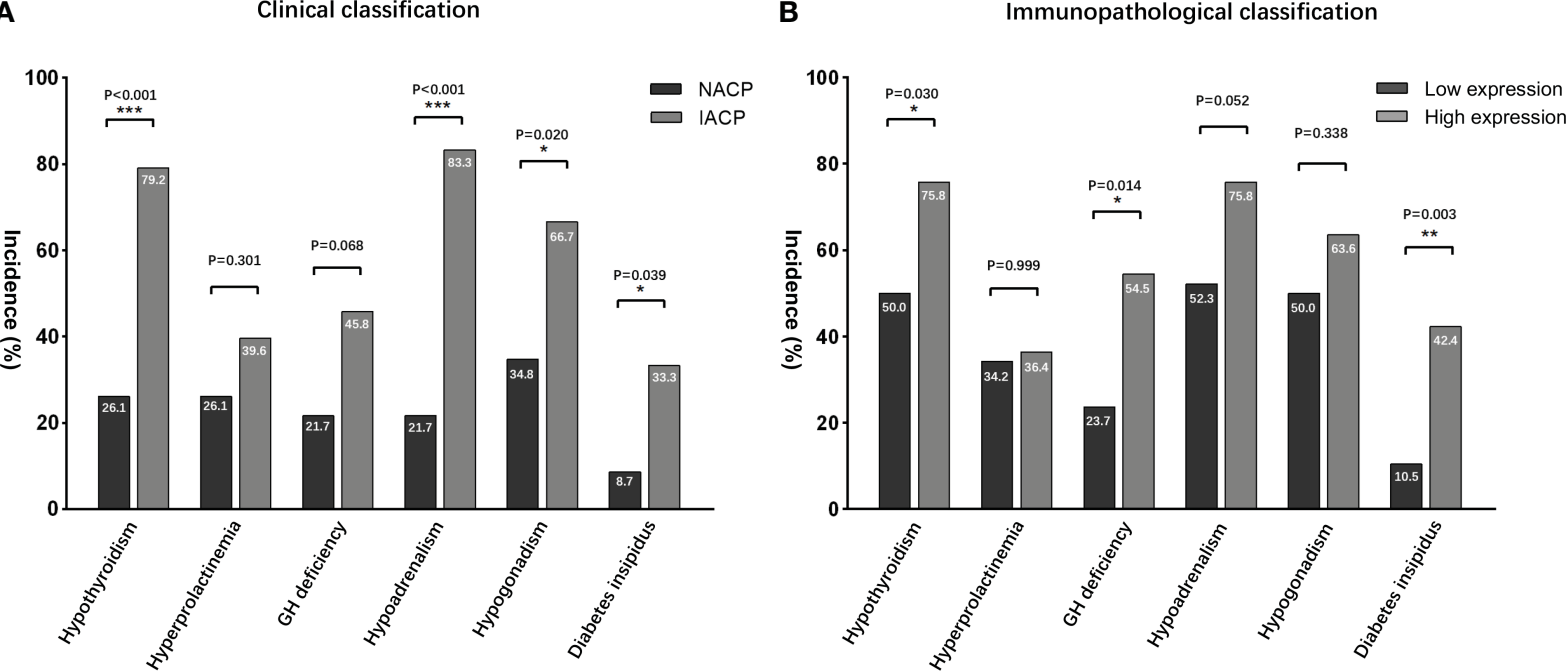
Postoperative endocrinological deficit comparisons. **(A)** According to the radiological classifications, IACPs developed a higher incidence of hypopituitarism NACPs; **(B)** similar results were found in the high TGF-β1 expression group based on the immunopathological classification. NACPs, non-invasive adamantinomatous craniopharyngiomas. *, p<0.05, **, p< 0.01, ***, p<0.001.

Based on the H-score mean value in the immunohistochemical analysis, the primary patients were divided into groups of high and low expression levels (**Figure 3**; **Supplemental Figure 1**). Preoperatively, no significant difference in endocrinopathy between the low and high expression of the TGF-β1 groups was found when comparing the deficient hypothalamic–pituitary axis separately (**Table 5**). Postoperatively, compared with the NACP and low TGF-β1 expression group, patients with hypothalamic invasion and a high level of TGF-β1 expression had a higher percentage of hypopituitarism. Specifically, for factors of hypothalamic invasion, the significantly different endocrine axes included hypothyroidism (26.1% vs. 79.2%, *P* < 0.001), hypoadrenalism (21.7% vs. 83.3%, *P* < 0.001), and hypogonadism (34.8% vs. 66.7%, *P* = 0.02) and DI (8.7% vs. 33.3%, *P* = 0.039) (**Figure 4A**). Additionally, the high expression of TGF-β1 was significantly related to hypothyroidism (50.0% vs. 75.8%, *P* = 0.03), GH deficiency (23.7% vs. 54,5%, *P* = 0.014), and DI (10.5% vs. 42.4%, *P* = 0.003) (**Figure 4B**).

**Table 5:** Correlations of the expression levels for immunopathological biomarkers with clinical parameters in primary patients.

	TGF-β1			SMAD3			SMAD2			β-Catenin		
	Low	High	*P*	Low	High	*P*	Low	High	*P*	Low	High	*P*
General features												
Age (mean, years)	39.4	40.7	0.72	40.3	39.6	0.85	39.8	40.1	0.95	38.5	41.7	0.39
Gender (female)	24 (63.2%)	15 (45.5%)	0.16	23 (63.9%)	16 (45.7%)	0.16	13 (48.2%)	26 (59.1%)	0.46	21 (53.9%)	18 (56.3%)	0.99
Endocrinopathy												
Preop (mean)	0.9	1.3	0.18	0.8	1.4	**0.03**	1.0	1.1	0.82	1.1	1.2	0.77
Postop (mean)	2.1	3.0	**0.01**	2.6	2.3	0.52	2.2	2.6	0.40	2.4	2.5	0.85
Radiography												
Content (solid/cystic/mixed)	12/13/13	7/14/12	0.59	10/12/14	9/15/11	0.69	6/10/11	13/17/14	0.70	14/12/13	5/15/12	0.13
Calcification	25 (65.8%)	20 (60.6%)	0.81	24 (66.7%)	21 (60.0%)	0.63	17 (63.0%)	28 (63.6%)	0.45	24 (61.5%)	21 (65.6%)	0.81
Volume (mean, cm^3^)	10.6	17.6	**0.05**	10.7	17.2	0.06	9.2	16.8	**0.04**	10.6	18.0	**0.03**
Hypothalamic invasion												
Preop (Puget 0/I/II)	5/11/22	0/7/26	**0.05**	3/8/25	2/10/23	0.49	3/8/16	2/10/32	0.41	5/12/22	0/6/26	**0.03**
Postop (Vile 0/I/II)	16/20/2	7/13/13	**0.002**	11/17/8	12/16/7	0.94	11/12/4	12/21/11	0.41	17/14/8	6/19/7	0.06
BMI (kg/m^2^)												
Preop (mean)	24.5	25.3	0.46	25.1	24.6	0.63	24.7	25.0	0.81	25.2	24.5	0.54
Postop (mean)	24.8	28.7	**<0.001**	26.5	26.5	0.99	27.1	26.1	0.42	26.4	26.7	0.81
Treatment features												
GTR	36 (94.7%)	28 (84.9%)	0.24	32 (88.9%)	32 (91.4%)	0.99	24 (88.9%)	40 (90.9%)	0.99	35 (89.7%)	29 (90.6%)	0.99
PS preservation	19 (50.0%)	4 (12.1%)	**0.008**	15 (41.7%)	8 (22.9%)	0.13	10 (37.0%)	13 (29.6%)	0.60	20 (51.3%)	3 (9.4%)	**<0.001**

Bold values indicate significant differences of p < 0.05.

### Association between the expression levels of the TGF-β signaling pathway and clinical factors

The relationships between clinical parameters and the expression levels of TGF-β1, SMAD3, SMAD2, and β-catenin in the primary tumors are shown in **Table 5**. There were no statistically significant correlations between their expression levels and demographic features of age and gender. Similarly, no statistically significant differences between TGF-β1 signaling expression levels and radiological factors of calcification and content were found, but tumor volume was significantly correlated with the expression levels of TGF-β1, SMAD2, and β-catenin, with higher expression in larger volume (*P* = 0.05, *P* = 0.04, and *P* = 0.03, respectively). Additionally, based on the preoperative hypothalamus involvement classification of Puget grade, higher level expression levels of TGF-β1 and β-catenin were observed in tumors with more severe hypothalamic invasion (*P* = 0.05 and *P* = 0.03, respectively). Moreover, according to the postoperative hypothalamic damage assessment system developed by de Vile et al. (28), there was only a significant association revealed between TGF-β1 expression level and hypothalamic damage extent due to tumor invasion (*P* = 0.002). It also demonstrated that TGF-β1 expression level was significantly associated with postoperative neurophysiological factors of endocrinopathy and BMI (*P* = 0.01 and *P* < 0.001, respectively), but not with their corresponding preoperative findings (*P* = 0.18 and *P* = 0.46, respectively).

### Predictors of recurrence/progression

**Figure 5:**
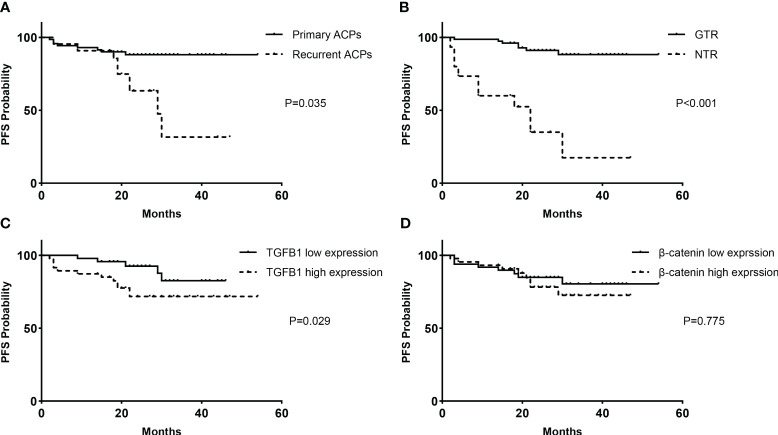
Kaplan–Meier survival functions. PFS analyses based on the classification of clinical factors for all cases of primary and recurrent ACPs (93 cases): **(A)** treatment history and **(B)** extent of resection. PFS analyses according to immunopathological expression levels for primary ACPs (71 cases): **(C)** TGF-β1 and **(D)** β-catenin. PFS, progression-free survival.

Patients with recurrent tumors were more likely to experience tumor progression than patients with primary tumors (40.9% vs. 11.3%, *P* = 0.035) (**Figure 5A**). The extent of resection had a significant impact on the tumor recurrence/progression. Compared with progression-free cases, those who presented with recurrence during follow-up had a significantly lower percentage of GTR (94.7% vs. 41.2%, *P* < 0.001). Kaplan–Meier survival analysis showed that patients with GTR had significantly longer mean PFS than patients without GTR (49.9 vs. 21.7 months, *P* < 0.001) (**Figure 5B**). Furthermore, tumor progression was also significantly associated with the expression level of TGF-β1, with a remarkably higher percentage of recurrence in the high expression group (27.7% vs. 8.7%, *P* = 0.029) (**Figure 5C**), but not significantly associated with the expression level of β-catenin (16.3% vs. 20.5%, *P* = 0.775) (**Figure 5D**).

## Discussion

Craniopharyngiomas are rare dysplastic tumors of low biobehavioral malignancy arising along the congenital craniopharyngeal duct (29). Previous studies revealed that ACPs are driven by somatic mutations in *CTTNNB1* (encoding β-catenin) that affect β-catenin stability, leading to its predominantly cytoplasmic and nuclear accumulation and the persistent activation of the WNT pathway (30, 31). Although hypothalamic involvement and treatment-induced endocrinopathy were reported in previous studies about ACPs (32), the association between clinical factors and molecular expression levels has rarely been well illuminated. Our study found that the expression levels of TGF-β1 and SMAD3 were positively correlated with the aberrant nuclear accumulation of β-catenin. The recurrent ACPs had higher expression levels of β-catenin and TGF-β1 than the primary ones, and similar results were observed in the comparison between groups of patients with/without hypothalamic invasion among primary tumors. Such differences in expression might indicate an important molecular mechanism contributing to the distinct tumor growth pattern and invasive ability of ACPs. Our study also found that surgery-induced endocrinopathy was associated with the morphological invasion of the hypothalamus and the molecular high expression of TGF-β1. Finally, the extent of resection, treatment history, hypothalamic invasion, and the levels of TGF-β1 expression had significant effects on tumor recurrence/progression after expanded EEA surgery separately.

According to the literature, endocrinological dysfunction after surgery for craniopharyngiomas has always been troublesome due to its original site and growth pattern (33). Many studies have indicated that the factor of pituitary stalk infiltration by tumors could result in worse pituitary function after surgery despite its partial or complete preservation (34–36). Our study focused on another effect of hypothalamic invasion on postoperative endocrinopathy, and postoperative endocrinological status comparison revealed more severe hypopituitarism in patients with IACPs, including hypothyroidism, hypoadrenalism, hypogonadism, and DI. The reason may be related to the IACPs directly infiltrating and destroying the floor of the third ventricle, which inevitably leads to defects of the hypothalamus nuclei and damage to the hypothalamic function after surgery (37, 38). We also found a similar pattern of postoperative endocrinopathy according to the immunopathological classification, with more severe hormone deficiencies in the high TGF-β1 expression group as compared with the IACP group. Additionally, TGF-β1 expression was also positively associated with the level of postoperative BMI, which indicated a significant association with hypothalamus damage after surgery (32, 39). These findings suggested that TGF-β1 was correlated with poor postoperative hypothalamic function, which may be added as a pathological indicator for the prediction of prognosis.

Studies of human epithelial carcinomas, such as breast cancer, colon carcinoma, lung cancer, and prostatic adenocarcinoma, have shown that the TGF-β signaling pathway could promote cell epithelial–mesenchymal transition by a variety of mechanisms, including cell autocrine/paracrine mechanisms and coactivation with the WNT pathway, which were considered to be associated with more aggressive tumor behavior and poor prognosis (22, 40, 41). Our comparison between groups of IACPs and NACPs concluded that the biological behavior of hypothalamic invasion of ACPs might be correlated with the upregulation of the TGF-β signaling pathway, with higher expression of TGF-β1 and β-catenin compared with NACPs. Additionally, the recurrent ACPs revealed higher expression of TGF-β1 and overaccumulation of β-catenin than the primary ones. Our study also found a significant correlation between the expression of TGF-β1, SMAD3, and β-catenin, in line with previous findings that upregulation of the TGF-β–SMAD2/3 signaling pathway was positively correlated with the overactivation of the Wnt/β-catenin signaling pathway (42, 43), indicating a possible role of TGF-β1 in the regulation of β-catenin nuclear-cytoplasmic distribution in a reciprocal communication manner. These findings provided evidence that the TGF-β signaling pathway plays a potential role in the pathogenesis and development of ACPs.

Regarding the treatment outcomes, the present study revealed that the TGF-β1 expression level was negatively associated with the rate of pituitary stalk preservation and the postoperative TVF integrity in the primary cases. Studies have reported that protection of the two important structures was essential for improved postoperative pituitary function and quality of life (44, 45). However, the balance between the functional protection and extent of resection should be considered seriously when planning a surgical strategy, because, as our and previous studies revealed, the partial resection of ACPs significantly increased the likelihood of tumor recurrence/progression (46). Worse still, the recurrent patients experienced much more severe endocrinological defects, a lower percentage of GTR, and a higher probability of recurrence after EEA surgery than the primary cases. Instead, for tumors that have invaded the hypothalamus seriously, hypothalamus-sparing surgery with radio-oncological treatment strategies, typically with external beam radiotherapy using protons, is efficient in controlling progression and preventing neuropsychological sequelae (47, 48). Further efforts to improve treatment outcomes in patients with craniopharyngiomas should be made by the pattern of multidisciplinary cooperation (49), and our findings of the upregulation of TGF-β1 in the hypothalamic involvement and tumor progression may provide a clue for the molecular pathogenesis of aggressive biological behavior and poor prognosis of ACPs. Subsequent *in-vitro* or *in-vivo* studies with scientific initiatives aiming at the TGF-β signaling pathway should be carried out to improve our understanding of the molecular pathogenesis of ACPs, with the perspective of developing potential targeted therapies against hypothalamus invasion and tumor progression (50).

## Conclusion

The present study found that the expression levels of TGF-β1 and β-catenin were positively correlated and their overexpression was closely associated with recurrent ACPs. Consistent with the clinicopathological classification of hypothalamus invasion, the immunopathological phenotypes of TGF-β1 expression for ACPs may provide more evidence for the prediction of postoperative pituitary dysfunction and poor prognosis after EEA surgery. A precise basic scientific investigation of the mechanism of the TGF-β signaling pathway in APCs needs to be conducted in the future.

## Data availability statement

The raw data supporting the conclusions of this article will be made available by the authors, without undue reservation.

## Ethics statement

This study was approved by the Ethics Committee of Beijing Tiantan Hospital (approval number KY 2021-041-02), and written informed consent was obtained from all patients. The studies were conducted in accordance with the local legislation and institutional requirements. The participants provided their written informed consent to participate in this study.

## Author contributions

LJ and SG designed the research. KC, WW, YX, NQ, FL, and SR performed the basic research. LC, HZ, JB, CZL, CHL, and PZ performed the surgery and data collection. LJ performed the data analysis and wrote the paper. YZ and SG critically revised the paper. All authors contributed to the article and approved the submitted version.

## Supplementary material

Supplementary Figure 1 Other two illustrated cases of IHC staining images of low and high expression of TGFB1, SMAD2, SMAD3, and β-catenin (x400). IHC = immunohistochemical staining.
