# Supplementary figures and images for: Correlations between the expression of molecules in the TGF-β signaling pathway and clinical factors in adamantinomatous craniopharyngiomas
Source: Front Endocrinol (Lausanne). 2023 Oct 3;14:1167776. doi: 10.3389/fendo.2023.1167776 (PMC10579895; doi:10.3389/fendo.2023.1167776)

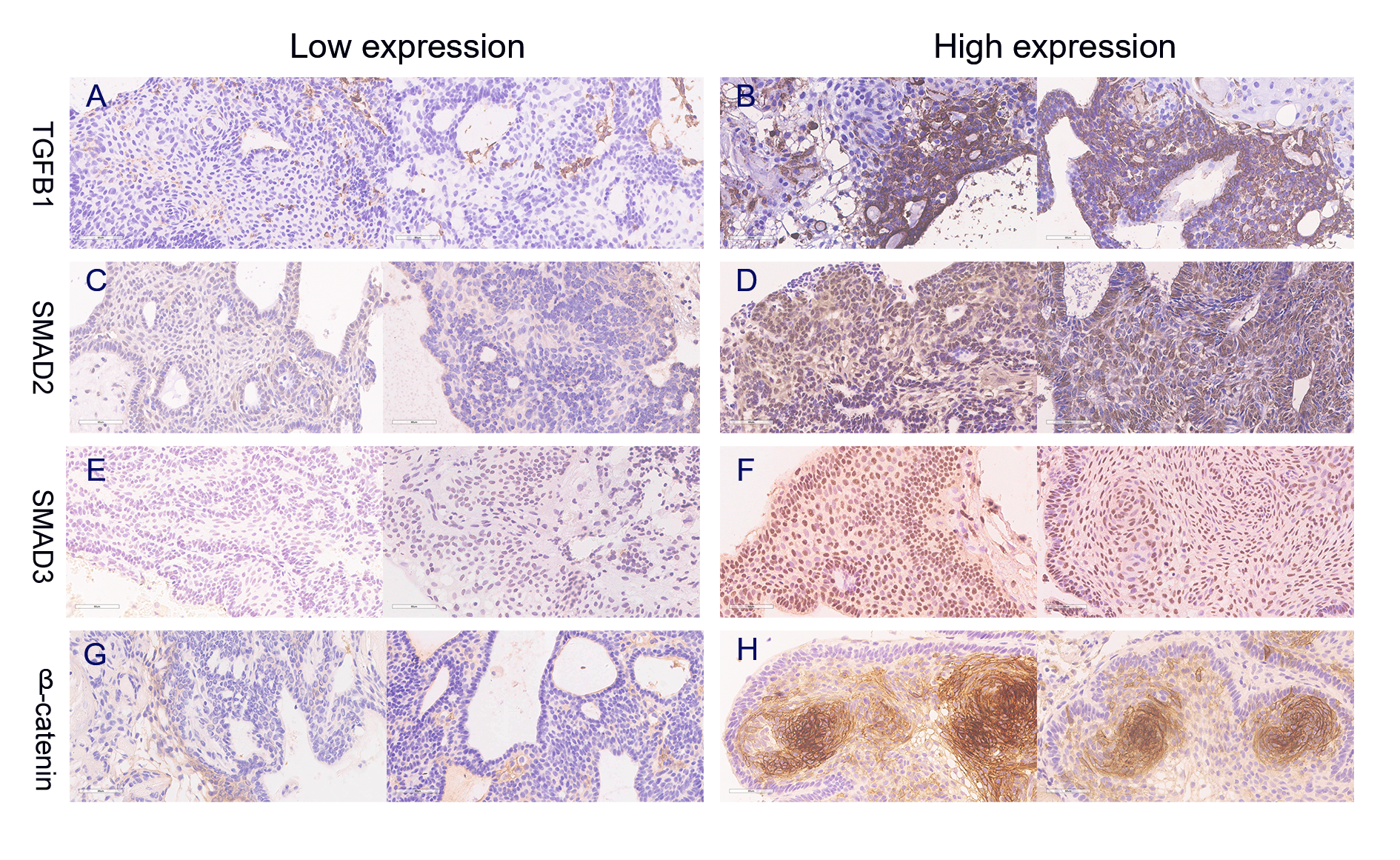

Supplement: Supplementary Figure 1 — Other two illustrated cases of IHC staining images of low and high expression of TGFB1, SMAD2, SMAD3, and β-catenin (x400). IHC = immunohistochemical staining. [file Image_1.tif]
